# Supplementary material for: Discovery of Nosiheptide, Griseoviridin, and Etamycin as Potent Anti-Mycobacterial Agents against Mycobacterium avium Complex
Source: Molecules. 2019 Apr 16;24(8):1495. doi: 10.3390/molecules24081495 (PMC6514863; doi:10.3390/molecules24081495)
Supplement: Supplementary file 1 [file molecules-24-01495-s001.pdf]

Supplementary Material

General Experimental Procedures

ESI-MS spectrometry was conducted on a JMS-T100LP spectrometer (JEOL, Tokyo, Japan). UV and IR spectra were measured with a U-2800 spectrophotometer (Hitachi, Tokyo, Japan) and FT/IR-460 plus spectrometer (JASCO, Tokyo, Japan), respectively. The ^{13}C-NMR and ^{1}H-NMR spectra of **1–3** were taken on the XL-400 NMR system (Agilent, Santa Clara, CA, USA). Samples were measured in $CHCl_3$-*d* : MeOH-d_4 = 9 : 1 for **1**, dimethyl sulfoxide-d_6 (DMSO-d_6) for **2**, and $CHCl_3$-*d* for **3**. The solvent peak was used as an internal standard at 7.26 ppm for $CHCl_3$-*d* : MeOH-d_4 = 9 : 1, 2.48 ppm for DMSO-d_6, and 7.26 ppm for $CHCl_3$-*d* for the ^{1}H NMR spectral data, and 77.0 ppm for $CHCl_3$-*d* : MeOH-d_4 = 9 : 1, 39.5 ppm for DMSO-d_6, and 77.0 ppm for the ^{13}C NMR spectral data.

*S-1. Structural elucidation of **1–3***

S-1.1. Compound **1**

Compound **1** was identified as nosiheptide by comparison with an authentic sample [1]. As shown in Figure S1, the ^{1}H-NMR spectra of **1** had good agreement with an authentic sample. Furthermore, UFLC analysis was performed using a Prominence UFLC system (SHIMADZU) with a connected Shin pack XR-ODS column (SHIMADZU) under the following conditions: mobile phase, 7-min gradient from 30% CH_3CN to 70% CH_3CN containing 0.1% H_3PO_4; flow rate, 0.55 mL/min; detection, UV at 210 nm; column temperature, 50 °C; injection volume, 0.2 µg (0.1 mg/mL, 2 µL, in 1% DMSO). The authentic sample and natural product **1** were eluted as a peak with a similar retention time (Figs. S2a and S2b), and each peak overlapped when a mixture of equal parts was analyzed (Fig. S2c).

Compound **1**: ^{1}H-NMR (400 MHz, $CHCl_3$-*d* : MeOH-d_4 = 9 : 1) δ = 1.15 (d, 3H, J = 5.5 Hz), 1.67 (d, 3H, J = 7.0 Hz), 2.02 (br, 1H), 2.36 (t, 1H, J = 12.9 Hz), 2.44 (s, 3H), 3.24 (obscured, 1H), 3.65 (m, 1H), 4.01 (m, 1H), 4.12 (d, 1H, J = 11.7 Hz), 4.42 (s, 1H), 5.02 (d, 1H, J = 10.5 Hz), 5.57 (d, 1H, J = 1.6 Hz), 5.75 (t, 1H, J = 9.8 Hz), 5.83 (d, 1H, J = 11.3 Hz), 5.96 (br, 1H), 6.31 (q, 1H, J = 7.0 Hz), 6.58 (d, 1H, J = 1.6 Hz), 7.11 (d, 1H, J = 7.0 Hz), 7.39 (t, 1H, J = 7.4 Hz), 7.48 (d, 1H, J = 10.5 Hz), 7.62 (s, 1H), 7.65 (s, 1H), 7.79 (br, 1H), 7.90 (s, 1H), 7.94 (br, 1H), 8.16 (s, 1H), 8.27 (s, 1H), 8.45 (s, 1H); HR ESI-MS (*m/z*) $[M + H]^+$ found: 1222.1540, calculated: 1222.1556 for $C_{51}H_{44}N_{13}O_{12}S_6$.

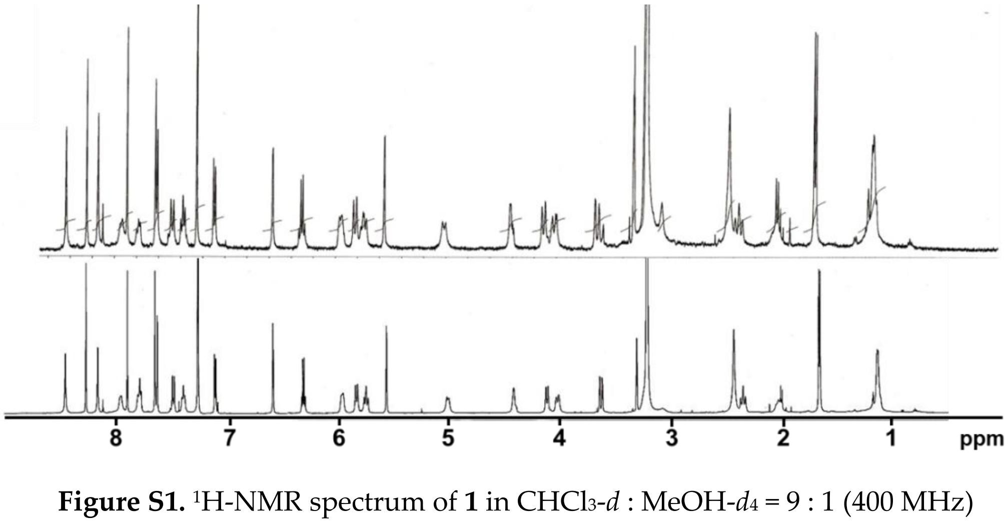

Figure S1. ^{1}H-NMR spectrum of **1** in $CHCl_3$-*d* : MeOH-d_4 = 9 : 1 (400 MHz)

Upper data represents ^{1}H-NMR spectrum of the natural product. Lower data represents that of the authentic sample of nosiheptide.

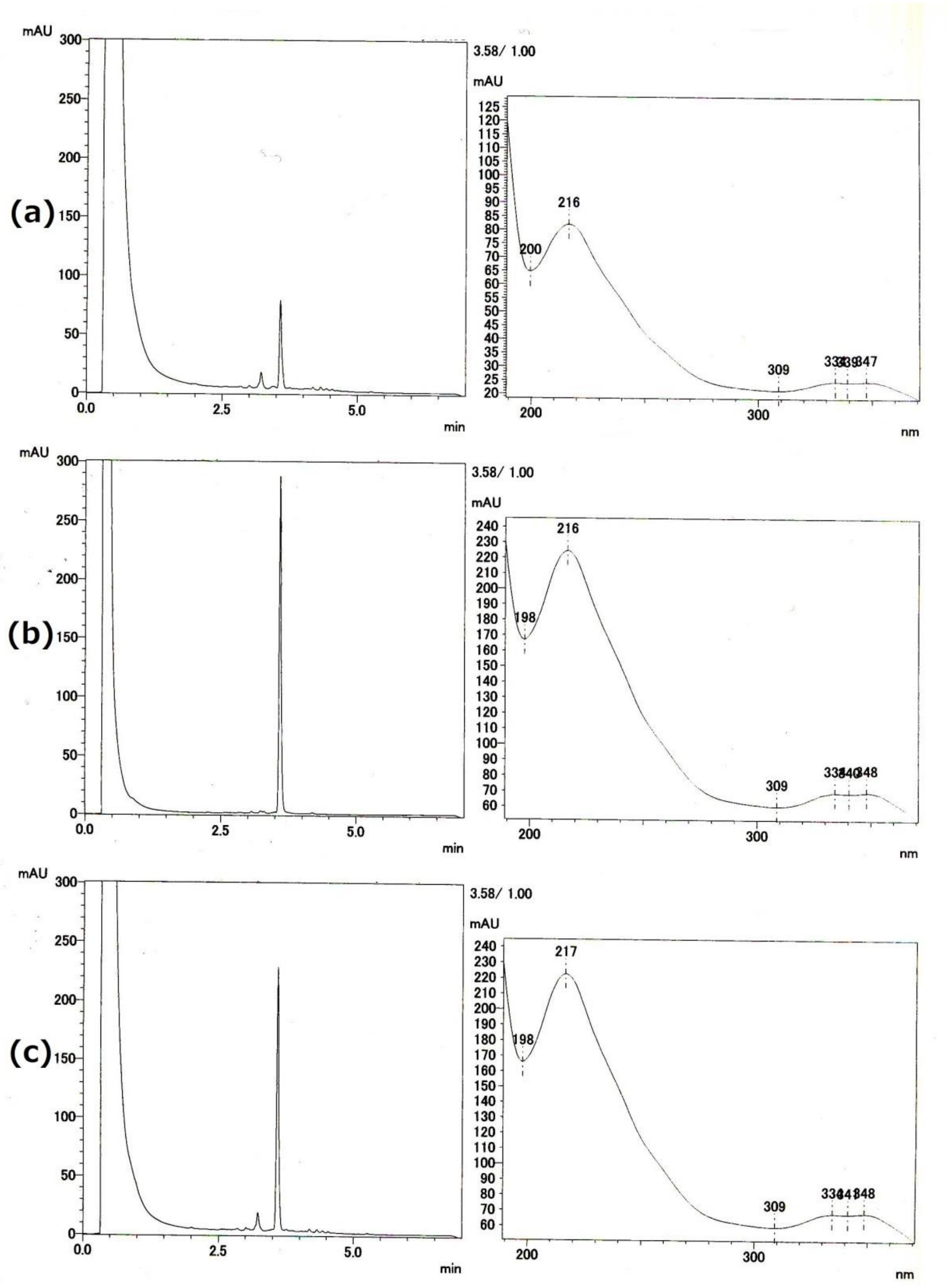

Figure S2. Comparison of the natural product and authentic sample of nosiheptide

(a): Natural product. (b): Authentic sample. (c): Mixture of the natural product **1** and authentic sample.

S-1.2. Compounds **2** and **3**

Compounds **2** and **3** were identified as griseoviridin and etamycin (viridogrisein) by comparison with the reported chemical shift values by ^{1}H-NMR and ^{13}C-NMR spectra, respectively [2].

Compound **2**: ^{1}H-NMR (400 MHz, DMSO-d_6) and ^{13}C-NMR (100 MHz, DMSO-d_6) see Table S1; HR ESI-MS (*m*/*z*) [M + Na]$^{+}$ found: 500.1470, calculated: 500.1467 for $C_{22}H_{27}N_3NaO_7S$.

Compound **3**: ^{1}H-NMR (400 MHz, CHCl$_3$-*d*) δ = 0.60 (d, 3H, J = 7.0 Hz), 0.77 (d, 3H, J = 7.0 Hz), 0.94 (overlapped, 3H), 0.96 (overlapped, 3H), 0.98 (overlapped, 3H), 1.17 (d, 3H, J = 6.6 Hz), 1.39 (d, 3H, J = 6.2 Hz), 1.48 (m, 1H), 1.80 (m, 1H), 1.84 (m, 1H), 1.91 (m, 1H), 2.06 (d, 1H, J = 14.5 Hz), 2.15 (m, 1H), 2.21 (m, 1H), 2.79 (s, 3H), 2.82 (s, 3H), 2.92 (s, 3H), 3.73 (dd, 1H, J = 11.0 Hz, 5.9 Hz), 3.87 (d, 1H, J = 16.8 Hz), 4.42 (dd, 1H, J = 11.0 Hz, 6.2 Hz), 4.54 (m, 1H), 4.88 (m, 1H), 4.89 (m, 1H), 5.06 (overlapped, 1H), 5.08 (overlapped, 1H), 5.17 (overlapped, 1H), 5.18 (overlapped, 1H), 5.35 (d, 1H, J = 16.8 Hz), 5.66 (s, 1H), 6.69 (d, 1H, J = 11.7 Hz), 7.25 (overlapped, 1H), 7.26 (overlapped, 1H), 7.32 (overlapped, 1H), 7.41 (overlapped, 3H), 8.07 (d, 1H, J = 3.5 Hz), 8.33 (d, 1H, J = 8.6 Hz), 8.95 (d, 1H, 7.4 Hz), 11.78 (s, 1H); ^{13}C-NMR (100 MHz, CHCl$_3$-*d*) δ = 8.6, 13.6, 15.5, 18.2, 21.2, 21.7, 23.2, 24.4, 28.6, 30.2, 32.0, 35.7, 35.9, 37.7, 39.6, 46.1, 49.1, 52.6, 53.3, 54.2, 58.4, 58.7, 63.0, 70.3, 70.8, 125.9, 128.7, 129.2, 129.2, 129.8, 130.7, 130.9, 139.9, 157.6, 166.1, 167.5, 167.9, 169.2, 169.8, 172.4, 173.5, 174.1 (Only the major rotamer is shown); HR ESI-MS (*m*/*z*) [M + Na]$^{+}$ found: 901.4468, calculated: 901.4435 for $C_{44}H_{62}N_8NaO_{11}$.

Table S1. The ^{1}H-NMR and ^{13}C-NMR data of **2** and reported values of griseoviridin in DMSO-*d*6.

	**2**		**Reported values [2]**	
**position**	δ_C[a]	δ_H[b] mult (*J* in Hz)	δ_C[c]	δ_H[d] mult (*J* in Hz)
**1**				
**2**	130.4, q		130.4, q	
**3**	144.6, t	7.32, dd (9.0, 7.4)	144.4, t	7.35, dd (9.0, 7.5)
**4**	37.4, d	2.97, 2.39, m	37.3, d	2.89, m, 2.40, dd (7.5)
**5**	70.8, t	5.10, m	70.7, t	5.12, dq
**6**				
**7**	170.7, q		170.6, q	
**8**	50.2, t	4.52, m	50.1, t	4.53, ddd (7.5, 10.5, 5.0)
**9**	38.4, d	3.44, 2.66, m	38.3, d	3.47, m, 2.67, dd (11, 14.5)
**10**	NH	7.20, d (8.6)	NH	
**11**	158.8, q		158.7, q	
**12**	134.4, q		134.3, q	
**13**				
**14**	162.7, q		162.1, q	
**15**				
**16**	141.3, t	8.55, s	144.1, t	8.56, s
**17**	35.5, d	2.86, 2.81, m	35.4, d	2.90, dd (9.5, 16.5), 2.81, dd (9.0, 16.5)
**18**	65.2, t	3.91, m	65.2, t	3.92, m
**19**	44.3, d	1.56, 1.45, m	44.2, d	1.57, m, 1.46, t (11)
**20**	69.1, t	4.1, m	68.9, t	4.11, m
**21**	136.5, t	5.52, dd (14.9, 8.2)	136.4, t	5.53, dd (15.5, 8.5)
**22**	130.1, t	6.18, dd (15.3, 10.6)	130.0, t	6.19, dd (15, 10.5)
**23**	128.4, t	5.95, dd (15.3, 10.6)	128.4, t	5.96, dd (15, 10.5)
**24**	129.3, t	5.71, dt (15.3, 3.9)	129.2, t	5.74, dt (15.5, 4.0)
**25**	40.2, d	3.91, 3.71, m	40.0, d	3.96, m, 3.77, m
**26**	NH	8.30, t (5.87)	NH	
**27**	162.1, q		162.6, q	
**28**	20.3, s	1.37, d (6.3)	20.1, s	1.38, d (6.5)

[a]: 100 MHz. [b]: 400 MHz. [c]: 125 MHz. [d]: 500 MHz.

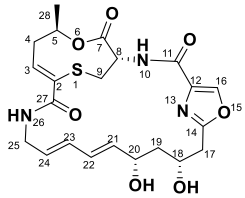

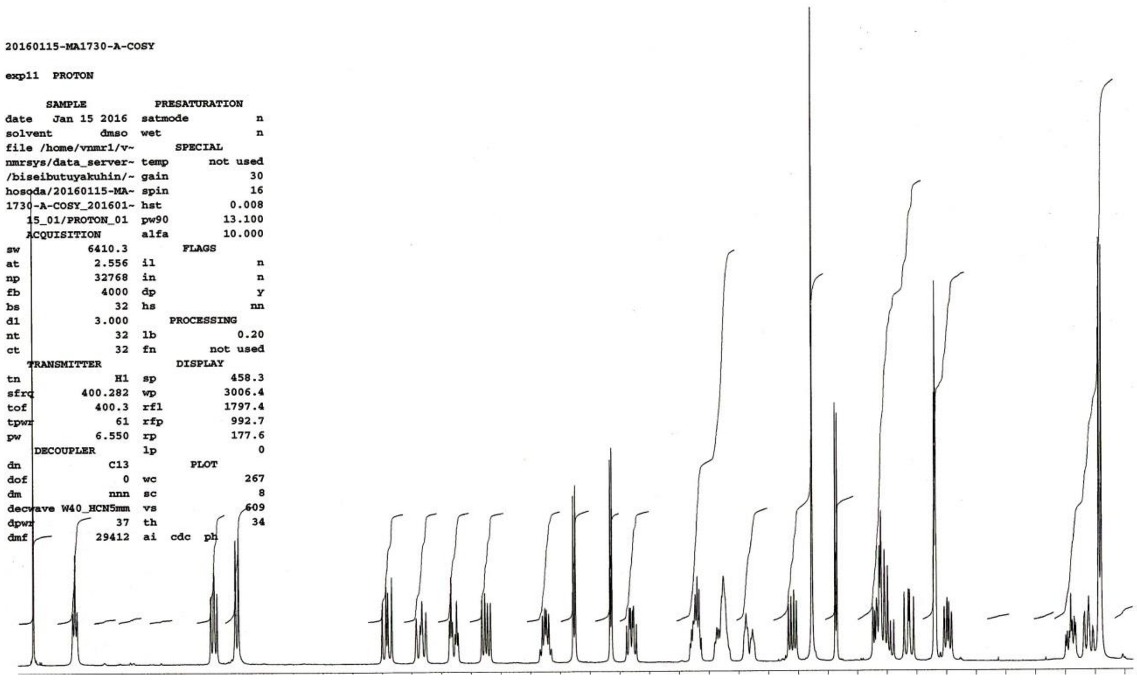

Figure S3. ^{1}H-NMR spectrum of **2** in DMSO-d_6 (400 MHz)

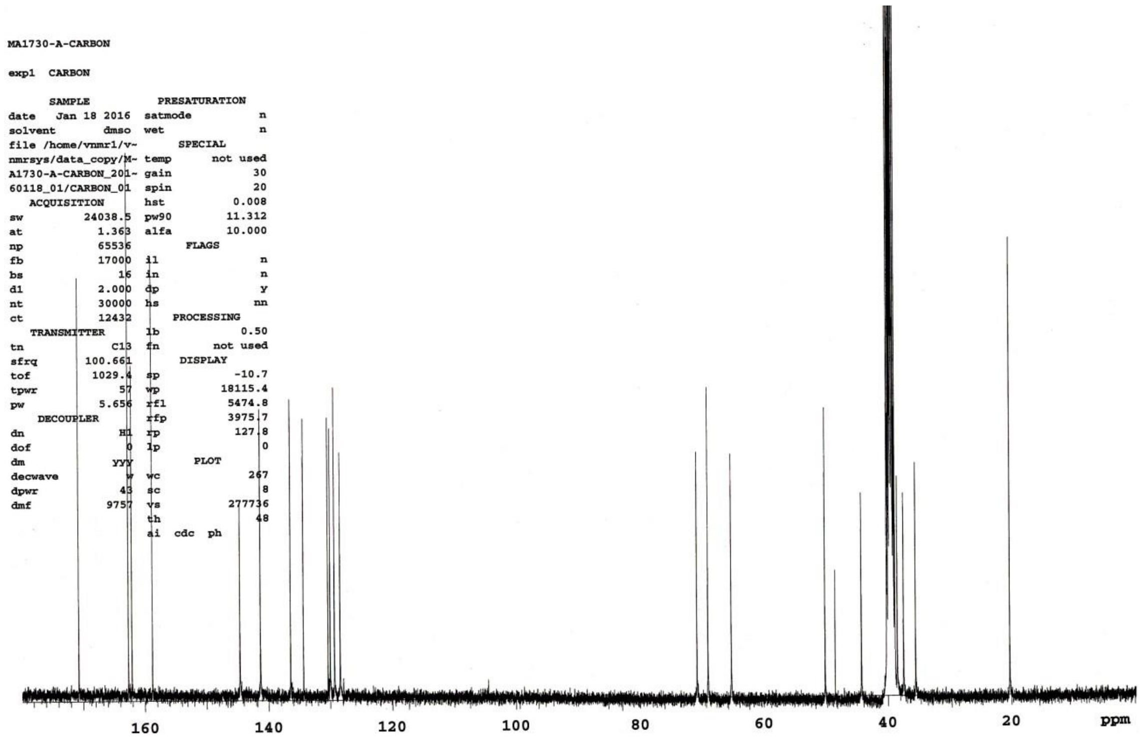

Figure S4. ^{13}C-NMR spectrum of **2** in DMSO-d_6 (100 MHz)

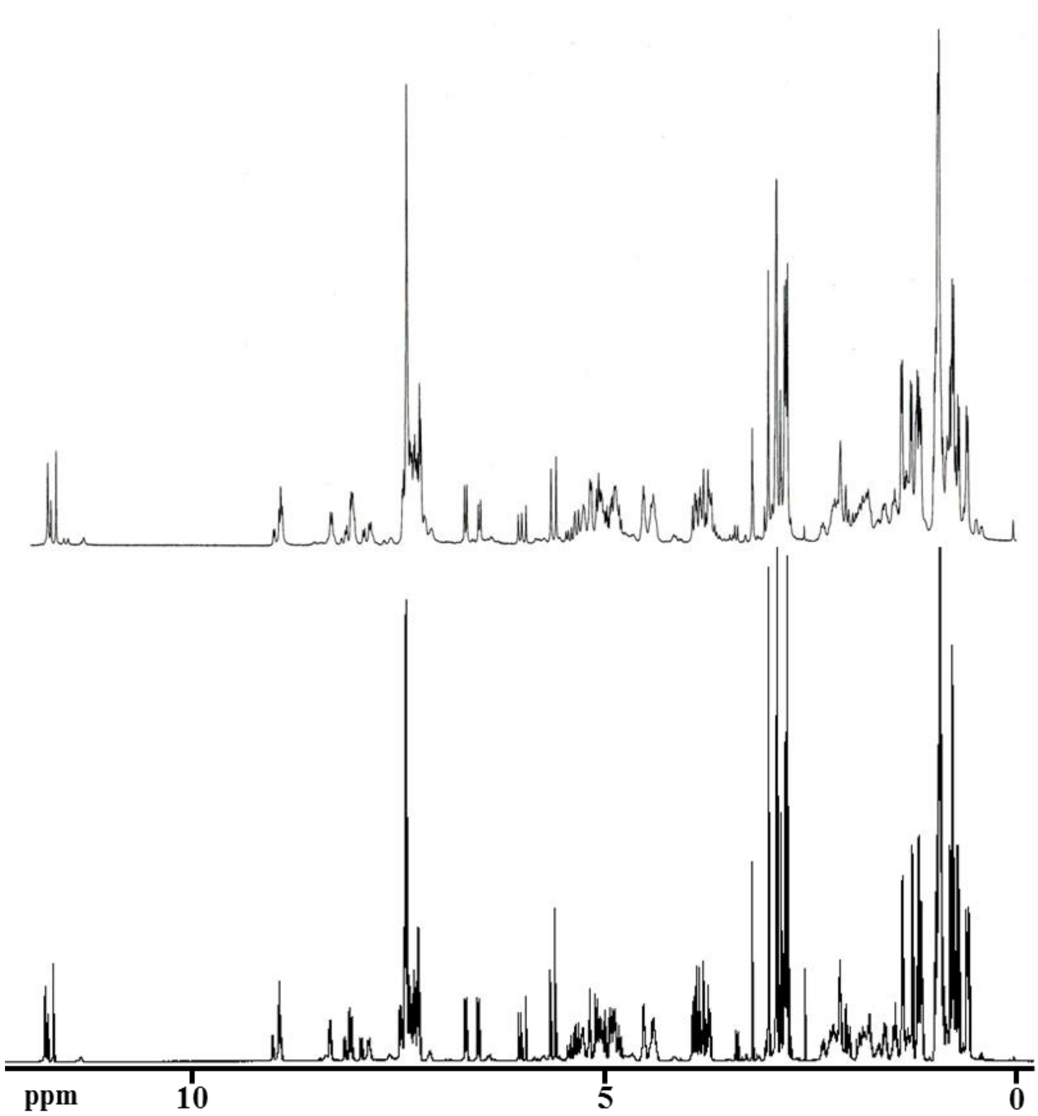

Figure S5. ^{1}H-NMR spectrum of **3** in $CHCl_3$-*d*

Upper data represents the ^{1}H-NMR (400 MHz) spectrum of **3**. Lower data represents the reported ^{1}H-NMR (500 MHz) spectrum data of etamycin [3].

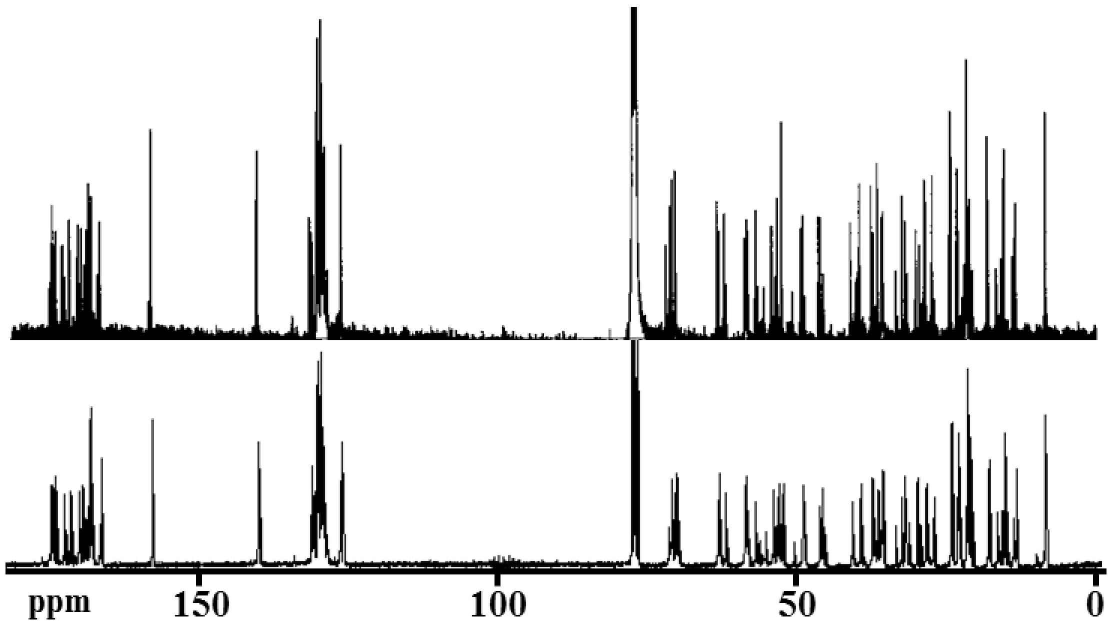

Figure S6. ^{13}C-NMR spectrum of **3** in $CHCl_3$-*d*

Upper data represents the ^{13}C-NMR (100 MHz) spectrum of **3**. Lower data represents the reported ^{13}C-NMR (125 MHz) spectrum data of etamycin [3].

Acknowledgments

We express our thanks to Prof. Hans-Dieter Arndt, School of Pharmacy, Friedrich-Schiller-Universität Jena, for providing authentic sample of nosiheptide.

References

1. Wojts, K.P.; Riedrich, M.; Lu, J.Y.; Winter, P.; Winkler, T.; Walter, S.; Arndt, H.D. Total synthesis of nosiheptide. *Angew. Chem. Int. Ed. Engl.* **2016**, *55*, 9772-9776, 10.1002/anie.201603140.
2. Xie, Y.; Li, Q.; Song, Y.; Ma, J.; Ju, J. Involvement of SgvP in carbon-sulfur bond formation during Griseoviridin biosynthesis. *Chembiochem.* **2014**, 15, 1183-1189, 10.1002/cbic.201400062.
3. Haste, N.M.; Perera, V.R.; Maloney, K.N.; Tran, D.N.; Jensen, P.; Fenical, W.; Nizet, V.; Hensler, M.E. Activity of the streptogramin antibiotic etamycin against methicillin-resistant Staphylococcus aureus. *J. Antibiot.* **2010**, *64*, 219-224, 10.1038/ja.2010.22.
